# Synthesis and Characterization of Waterborne Fluoropolymers Prepared by the One-Step Semi-Continuous Emulsion Polymerization of Chlorotrifluoroethylene, Vinyl Acetate, Butyl Acrylate, Veova 10 and Acrylic Acid

**DOI:** 10.3390/molecules22010184

**Published:** 2017-01-22

**Authors:** Hongzhu Liu, Jiming Bian, Zhonggang Wang, Chuan-Jin Hou

**Affiliations:** 1 Key Laboratory of Materials Modification by Laser, Ion and Electron Beams (Ministry of Education), School of Physics and Optoelectronic Technology, Dalian University of Technology, Dalian 116024, China; liuhz418@163.com; 2 School of Chemical Engineering, Dalian University of Technology, Dalian 116024, China; zgwang@dlut.edu.cn; 3 School of Light Industry and Chemical Engineering, Dalian Polytechnic University, Dalian 116034, China; 4 Post-Doctoral Research Station of Dalian Zhenbang Fluorocarbon Paint Stock Co., Ltd., Dalian 116036, China

**Keywords:** waterborne fluoropolymer, semi-continuous emulsion polymerization, synthesis

## Abstract

Waterborne fluoropolymer emulsions were synthesized using the one-step semi-continuous seed emulsion polymerization of chlorotrifluoroethylene (CTFE), vinyl acetate (VAc), *n*-butyl acrylate (BA), Veova 10, and acrylic acid (AA). The main physical parameters of the polymer emulsions were tested and analyzed. Characteristics of the polymer films such as thermal stability, glass transition temperature, film-forming properties, and IR spectrum were studied. Meanwhile, the weatherability of fluoride coatings formulated by the waterborne fluoropolymer and other coatings were evaluated by the quick ultraviolet (QUV) accelerated weathering test, and the results showed that the fluoropolymer with more than 12% fluoride content possessed outstanding weather resistance. Moreover, scale-up and industrial-scale experiments of waterborne fluoropolymer emulsions were also performed and investigated.

## 1. Introduction

Fluoropolymers used in high-performance coatings [1] have received much attention because of their unique construction [2], outstanding weatherability [3], and attractive surface properties [4,5]. They also have extensive applications in many fields, such as in the automotive industry [6], optic cables and microelectronics [7], plastics [8], woods [9], solar energy [10], and in the protection of cultural relics [11]. In recent years, research into waterborne fluoropolymer emulsions has attracted the attention of many investigators from the viewpoint of environmental protection and the shortage of resources.

Waterborne fluoropolymers are found in many categories, including water-emulsifying, water-thinned, and water-dispersible. Typically, water-dispersible products include aqueous dispersions of polytetrafluoroethylene (PTFE) [12,13,14], tetrafluoroethylene/hexafluoroethylene copolymer (FEP) [12], and tetrafluoroethylene/perfluoroalky vinyl ether copolymers (PFA) [12] for non-stick and anti-corrosion fields. However, those fluoropolymers are not necessarily suitable for use as conventional coating materials due to their high baking temperature and weak adhesion. Waterborne fluorinated polyacrylate [15,16,17] has also been used to a greater extent in surface coatings for paper, leather, and textile due to its characteristic water and oil repellency. Nevertheless, the cost of fluorinated acrylate monomers is comparatively high, where the increased use of the fluorinated monomers increases the prices of the coatings, and the weatherability of the coating is unsatisfactory, given that the fluoride atoms lie on the side chain of the polymer [11].

A fluoropolymer emulsion based on polyvinyldene fluoride (PVDF) using acrylic-modified fluoropolymer (AMF) latex technology is also reported [18,19,20]. The preparation of the product requires two stages: fluoropolymer emulsion polymerization and seeded acrylic emulsion polymerization. However, the homogeneity and storage stability of the emulsion needs be solved carefully, as it will greatly affect coating performances. The water-emulsifying or water-thinned fluoroethylene/vinyl ether (FEVE) fluoropolymer used for high-performance coatings is an alternating copolymer with a high regularity of chlorotrifluoroethylene (CTFE) and vinyl ether monomers [21,22]. However, vinyl ether monomers (i.e., hydroxybutyl vinyl ether, cyclohexane dimethanol vinyl ether) employed for FEVE copolymer are more expensive than ester-type monomers such as vinyl acetate, which hinders product acceptance by the market due to the high price of the coating.

In addition, different synthesis technologies [12,23,24,25,26,27,28] for water-based fluoropolymer emulsions are investigated for different products, including core–shell emulsion polymerization, the phase inversion emulsifying method, stepwise droplet technology, etc. Nevertheless, all of those methods cannot be finished by the one-step approach, involving special operation conditions and more process steps, which are not preferable selections for scale-up and industrialization of waterborne fluoropolymer emulsions.

For these reasons, a new polymerization technique for water-based fluoropolymers was developed to facilitate scale-up and industrialization. To our knowledge, the preparation method used in this paper has not been reported to-date.

In this study, a waterborne fluoropolymer was prepared by the one-step semi-continuous emulsion polymerization of a fluorine olefin monomer and an ester-type monomer. To accomplish this, the poisonous fluorine olefin monomer was added at once to the autoclave, and the remaining non-fluorine monomers were gradually added later. The feasibility of this method and the presented formulations were verified through scale-up and industrial-scale experiments. Through this method, waterborne fluoropolymers were readily prepared and characterized by IR (infrared spectrum), TG (thermal gravimetry) and DTG (differential thermal gravimetry), DSC (differential scanning calorimetry), and MFFT (minimum film-forming temperature). Meanwhile, the weatherability of the waterborne fluoropolymers were particularly evaluated by the QUV (quick ultraviolet) accelerated weathering testing.

## 2. Materials and Methods

### 2.1. Materials

CTFE, VAc (vinyl acetate), BA (*n*-butyl acrylate), Veova 10 (a vinyl ester of a highly branched decanoic acid) and AA (acrylic acid) were supplied from Dalian Zhenbang Fluorocarbon Paint Stock Co., Ltd. (Dalian, China) as polymerization grade monomers, and were used as received. NP-10 (octylphenol polyoxyethylene ether), 600#A (styrylphenol polyoxyethylene ether), and SDS (sodium dodecyl sulfate) were obtained from Liaoning Oxiran Chemstry Co., Ltd. (Liaoyang, China). KPS (potassium persulfate) and NaHCO_3_ (sodium bicarbonate) were purchased and used as the initiator and buffering agent, respectively. AMP-95 (2-amino-2-methyl-1-propanol) was purchased from Dow Chemicals (Midmland, MI, USA) and used as a pH adjusting agent.

### 2.2. Synthesis of the Waterborne Fluoropolymer

#### 2.2.1. Pre-Emulsification of the Monomers

The emulsifier mixture including NP-10 (4.63 g), 600#A (4.30 g), and SDS (0.99 g) was dissolved in deionized water (110.08 g). Next, VAc (149.98 g), BA (50.00 g), Veova 10 (50.00 g), and AA (3.90 g) were added to the above surfactant solution under 400 rpm stirring for 30 min to produce 373.88 g of the monomer emulsion.

#### 2.2.2. One-Step Semi-Continuous Polymerization

**Figure 1:**
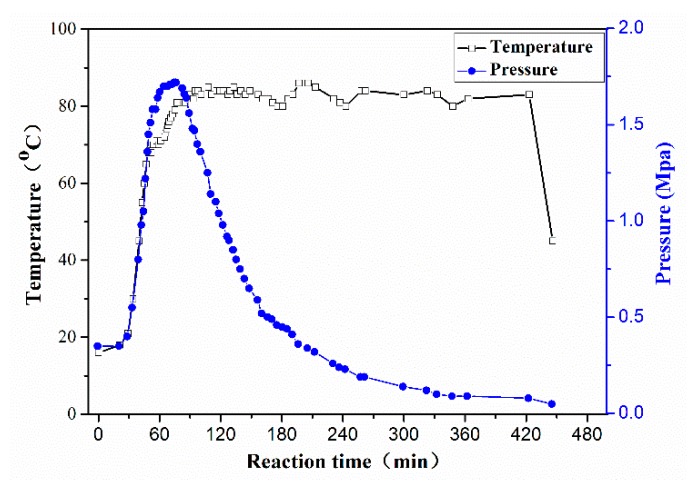
Change curve of pressure and temperature of polymerization (**P1**).

A mixed solution of water (30.54 g), an emulsifier mixture (1.29 g NP-10, 1.19 g 600#A, and 0.28 g SDS) and all of the CTFE (85.00 g) were introduced into a 1000 mL autoclave under vacuum. After homogenization at a rate of 500 rpm for 30 min, the monomer emulsion (21.80 g) described above, 0.35 g of KPS, 0.90 g of NaHCO_3_, and 175.25 g of deionized water were fed into the reactor. At a stirring rate of 500 rpm, the temperature in the autoclave was raised to 75 °C, and the mixture was reacted for 25 min; after that, the rest of the monomer emulsion (352.08 g) was mixed with KPS (1.16 g), and deionized water (60.04 g) was added dropwise at a constant rate for 3 h. After finishing the addition, KPS (0.35 g) dissolved in deionized water (18.25 g) was added to the autoclave. The polymerization was continued for an additional 3 h. The change of temperature and pressure of the reaction process with time in the reactor is shown in Figure 1. Finally, AMP-95 was added dropwise into the synthesized emulsion to control the pH of the emulsion in the range of 7–8. The amounts of the components used in the experiment are presented in detail in Table 1. The resulting waterborne fluoropolymer is denoted as **P1**.

**P2** was prepared by increasing the amount of CTFE to 130.00 g and reducing the amount of BA to 0 g in Table 1. **P3** and **P4** were obtained by adjusting the amount of CTFE to 50.00 g and 0 g, respectively, in Table 1 under the same polymerization conditions. For comparison, **P5** was prepared by intermittent emulsion polymerization (monomers and other materials were the same as Table 1). Amplification experiments based on the formulation in Table 1 (**P1**) at the 1000 L and 3000 L industrial scale were implemented, and are denoted as **P6** and **P7**.

### 2.3. Characterization

Non-volatiles were tested after drying at 110 °C for 2 h. Conversion (%) was calculated by the following formulas:

Conversion (%) = [W_1_ × Non-volatile (%) − W_2_]/W_3_ × 100%, (1)

where W_1_ is the total weight of all the materials in the autoclave, W_2_ is the weight of materials that cannot volatilize at drying, and W_3_ is the total weight of all monomers. The fluorine content of the emulsion was tested using the lanthanum nitrate method [29]. For the freeze/thaw stability test, 50 g of latex (in a 100 mL plastic bottle) was kept at −18 °C for 18 h. After another 6 h at room temperature, flocculation of the latex sample was observed. Next, 400 g of emulsion added into the enamel cup (1000 mL) was dispersed at a speed of 2500 r/min for 30 min. The damaged or flocculated emulsion was examined for its mechanical stability. The electrolyte stability was tested with 1 mL of CaCl_2_ solution (5%) added into a 10 mL test tube containing 5 mL of emulsion, with the delamination, precipitation, and flocculation observed after 1 h, 24 h, and 48 h.

The IR spectrum of the product was measured using pellets of the emulsion with an EQUINOX55 spectrometer (Bruker Optics, Karlsruhe, Germany) in the range of 400 to 4000 cm^−1^. The particle diameter and its distribution were measured using a Zetasizer-1000/DTS-5101 (Malvern Instruments, Malvern, UK). TG/DTG results were recorded by means of a NETZSCH TG 209 in the range of 40–800 °C at a heating rate of 10 °C/min under a nitrogen gas flow rate of 50 mL/min. The Tg value of the polymer was measured using a DSC Q2000 V24.9 Build121 (TA Instruments, New Castle, DE, USA) in the range of −30 to 250 °C with a heating rate of 10 °C/min under a nitrogen gas flow rate of 30 mL/min. The QUV accelerated weathering test was performed on the sample by an Accelerated Aging Tester (America Q-panel Company, Cleveland, OH, USA) under the conditions of 0.6 W/m^2^ irradiance, 60 °C base panel temperature, and 343 nm UV wavelength. The minimum film-forming temperature (MFFT) was determined by an MFFT meter (ZDT-1, 10–50 °C) according to GB 9267-2008. The molecular weight of the waterborne fluoropolymer emulsion was measured by gel permeation chromatography (GPC) analysis with a MAXIMA 820 GPC Analysis Report (Ventura, CA, USA), using a polystyrene calibration standard. Tetrahydrofuran (THF) was used as an eluent at a flow rate of 1 mL/min at 40 °C.

## 3. Results and Discussion

### 3.1. Emulsion Polymerization

The pre-emulsification of monomers **P1**, **P2**, **P3**, and **P4** was performed before the semi-continuous emulsion polymerization. **P5** was prepared by intermittent emulsion polymerization for comparison with **P1**–**P4**. All materials from Table 1 were fed into the autoclave and polymerized for 6 hours at 70–85 °C. The proportions of **P1** were used for the scale-up tests at 1000 L (**P6**) and 3000 L (**P7**). The experimental data from the reactions above are reported in Table 2, which showed promising results. The monomer conversion rates were quite high (≥96%), and the amounts of coagulum were minimal (≤0.1%). The appearance of all resultant emulsions was a milky liquid with a slight blue tint. The average particle diameters were 100–200 nm. The number average molecular weight (Mn) of the synthesized fluoropolymers was obtained to be 28,000–51,233 g·mol^−1^, with a wide molecular mole mass distributions (Mw/Mn). The results of the scale-up experiment (**P6**) and industrial-scale experiment (**P7**) proved that the method and formulations of the laboratory-scale experiment (**P1**) were stable, and that operation conditions could be achieved.

The seven resulting emulsions (**P1**–**P7**) were tested for their freeze/thaw, mechanical, and electrolyte stability. The **P5** sample showed poor stability in all categories, while the other samples exhibited better results. Therefore, the semi-continuous emulsion polymerization process was more effective in this study than the intermittent emulsion polymerization process.

### 3.2. Fourier Transform Infrared Spectroscopy (FT-IR) Analysis

**Scheme 1:**
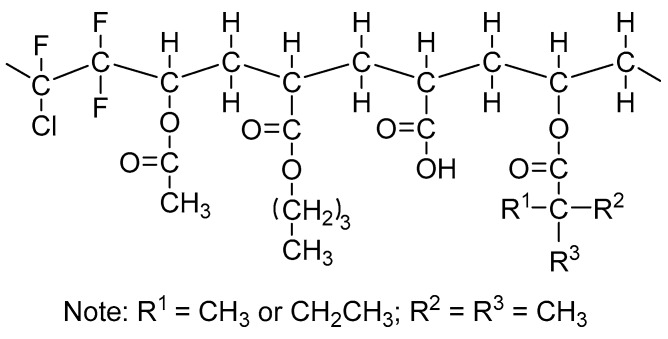
The structure of the waterborne fluorocarbon polymer.

**Figure 2:**
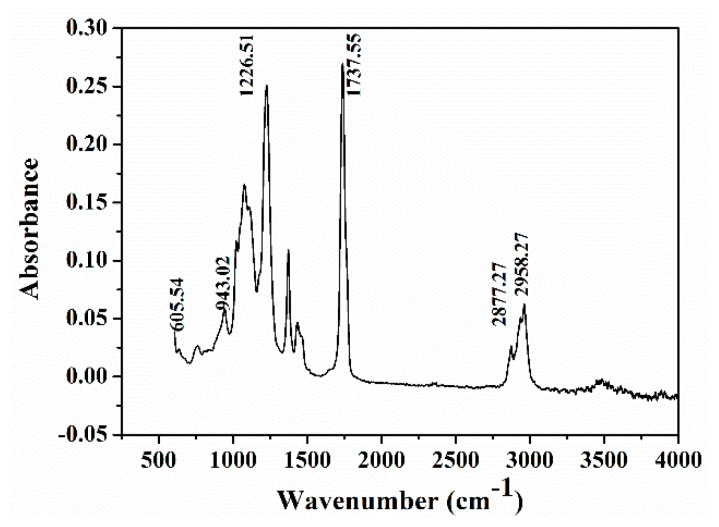
Fourier transform infrared (FT-IR) spectrum of the waterborne fluoropolymer of **P1**.

Waterborne fluorine emulsions undergo demulsification through CaCl_2_ solution (10%). After repeatedly washing the sediment collected with ethanol in order to remove residual monomers in the emulsion, the sediment was washed with water, and dried to a constant weight in a vacuum drying oven. The obtained sample was then used for IR analysis. The basic structure of the waterborne fluoropolymer is shown in Scheme 1, and the FT-IR spectrum of the fluoropolymer film **P1** is shown in Figure 2. The characteristic stretching peaks of CH_2_ and CH_3_ occurred at 2877.27 cm^−1^ and 2958.27 cm^−1^, respectively, and the stretching vibration of C=O at 1737.55 cm^−1^ was attributed to VAc, BA, Veova 10, and AA. As seen in the IR spectrum, the C-Cl stretching vibration occurred at 605.54 cm^−1^, the C-F stretching vibration occurred at 943.02 cm^−1^, and the C-F_2_ stretching vibration occurred at 1226.51 cm^−1^. The above three characteristic peaks revealed that CTFE could be well introduced into the emulsion particles as the desired monomer. The characteristic peak of the C=C (1650 cm^−1^) double bond was not found in the IR spectrum, indicating that there were no residual monomers in the sample.

### 3.3. Particle Diameter Distribution

**Figure 3:**
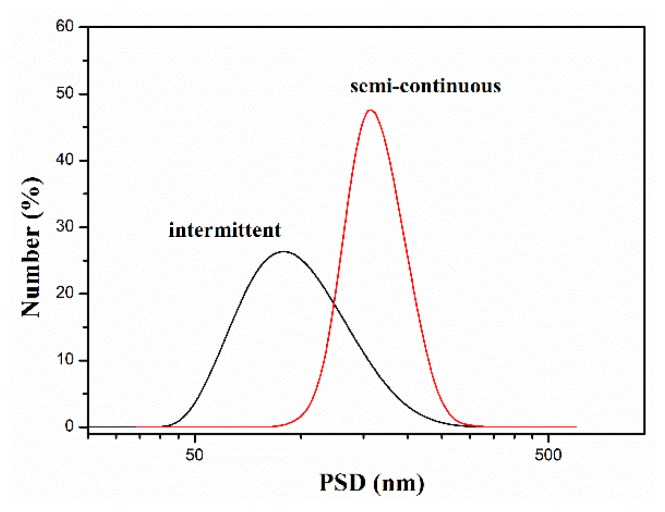
Effect of different emulsion polymerization methods on the particle diameter distribution (PSD).

The polymerization process had a significant influence on the particle diameter distribution (PSD). Adopting the formulation in Table 1, emulsions of **P1** and **P5** were synthesized by two different emulsion polymerization feeding methods: the semi-continuous droplet method, and the intermittent method. The test results of the emulsion particle diameter are shown in Figure 3.

As seen in Figure 3, emulsion **P1** had a large particle diameter ($\bar{D}$ = 163 nm) and narrow distribution, while emulsion **P5** had a small particle diameter ($\bar{D}$ = 101 nm) and wide distribution.

In the intermittent feeding method, all of the emulsifier was added at once into the reaction vessel, thus generating a larger number of micelles that kept the weight of all of the monomers constant, which led to a wide particle distribution and smaller particle diameter in the emulsion. For the semi-continuous droplet process, only a portion of emulsifier was added into the reaction vessel at the beginning of the reaction, leading to smaller micelles and fewer reaction centers, thus increasing the particle size.

**Figure 4:**
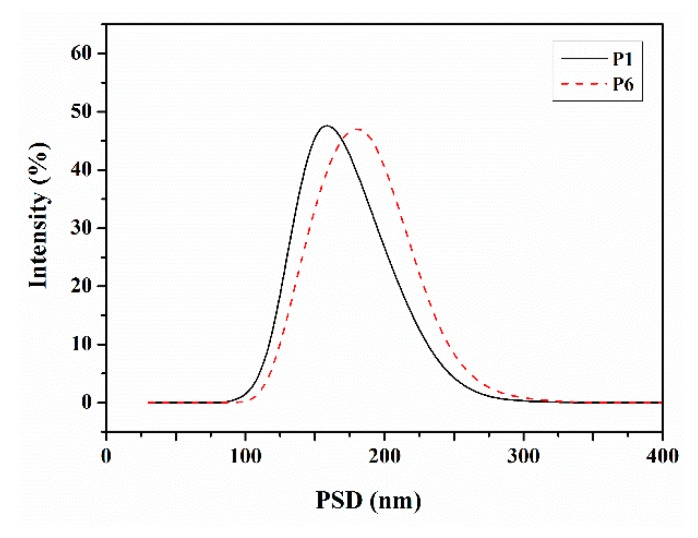
Comparison of the PSD in the laboratory-scale (**P1**) and industrial-scale experiments (**P6**).

Figure 4 shows the particle diameter distribution of the emulsions **P1** and **P6** prepared on a laboratory-scale and industrial-scale, respectively. We observed from Figure 4 that the average particle diameter ($\bar{D}$ = 163 nm) of emulsion **P1** was similar to that ($\bar{D}$ = 176 nm) of emulsion **P6**. These results showed that the emulsion product of scaling-up the experiment still has good reproducibility and stability with respect to the formulation and process.

### 3.4. Thermal Stability Analysis

**Figure 5:**
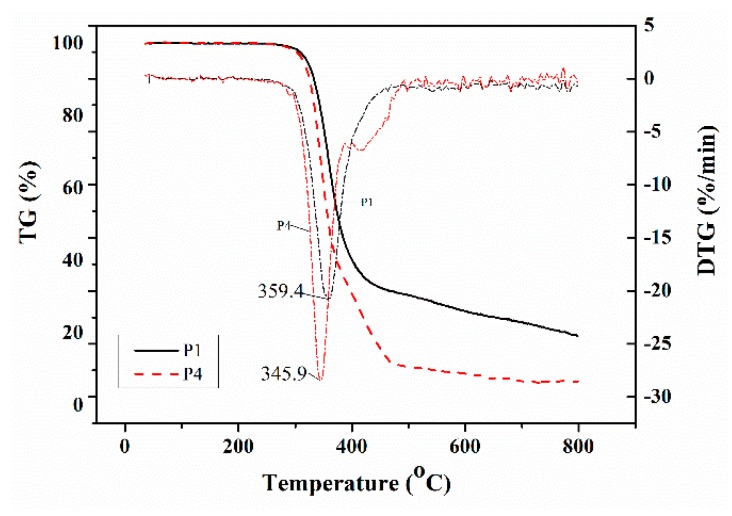
The thermal gravimetry (TG)/differential thermal gravimetry (DTG) curves of the waterborne fluoropolymer (**P1**) and the acrylic polymer (**P4**).

Approximately 2–5 mg of the fluoropolymer emulsion sample (**P1**), which was dried to a constant weight at 100 °C, was heated from 40 °C to 800 °C at a heating rate of 10 °C/min and at a speed of 40 mL/min ventilation with N_2_ gas. This was then compared with the acrylic emulsion sample (**P4**). The TG (thermal gravimetry) and DTG (differential thermal gravimetry) curves of the two types of polymer are shown in Figure 5. Figure 5 shows that the weightlessness rates of the fluoropolymer and acrylic polymer were both less than 1% under 250 °C and less than 2% under 300 °C, while the decomposition temperature of the waterborne fluoropolymer was higher than that of the acrylic polymer, where the former began to decompose at 292.3 °C, and the latter began to decompose at 272.9 °C. The largest decomposition rate of **P1** was at 359.4 °C, which was at a higher temperature than that of **P4** (345.9 °C). The TG analysis was repeated three times, and showed small errors and reliable reproducibility. According to the parallel tests (**P1** and **P4**), the temperature error of initial temperature of the lost weight and the rapid lost weight is less than ±2 °C [30,31]. Thus, the waterborne fluoropolymer had better heat stability than the fluorine-free acrylic emulsion. This phenomenon could be explained as follows: due to the introduction of C-F bonds in the structure of the copolymer, the groups containing the high bond energy C-F bonds could shield and protect the non-fluorinated segments below, thus improving the thermal stability of the fluoropolymer film [32].

### 3.5. Differential Scanning Calorimetry Analysis

**Figure 6:**
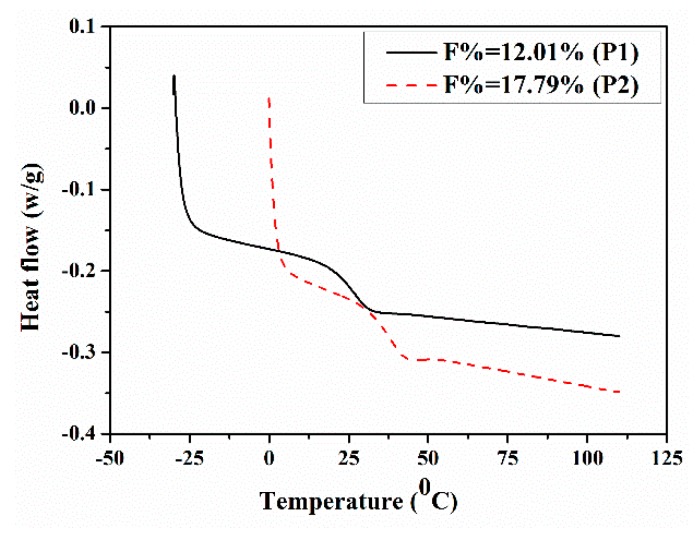
Differential scanning calorimetry (DSC) curves of fluoropolymers **P1** and **P2**.

The differential scanning calorimetry (DSC) curves of waterborne fluoropolymers **P1** and **P2** are shown in Figure 6. As can be seen in the two diagrams, fluoropolymer **P1** began to soften at 19.95 °C, and completely softened at above 31.11 °C. The tangent point (Tg) of the two baselines was identified as the polymer glass transition temperature, which was approximately 26.50 °C. Meanwhile, fluoropolymer **P2** began to soften at 31.70 °C and completely softened at above 42.37 °C, and the Tg of fluoropolymer **P2** was 31.11 °C. Calculating the Tg using the Fox equation gave 16.52 °C and 34.79 °C for fluoropolymers **P1** and **P2**, respectively. The Tg of fluoropolymer **P2** was higher than that of fluoropolymer **P1**, due to the different fluoride contents.

### 3.6. Film-Forming Characteristics

**Figure 7:**
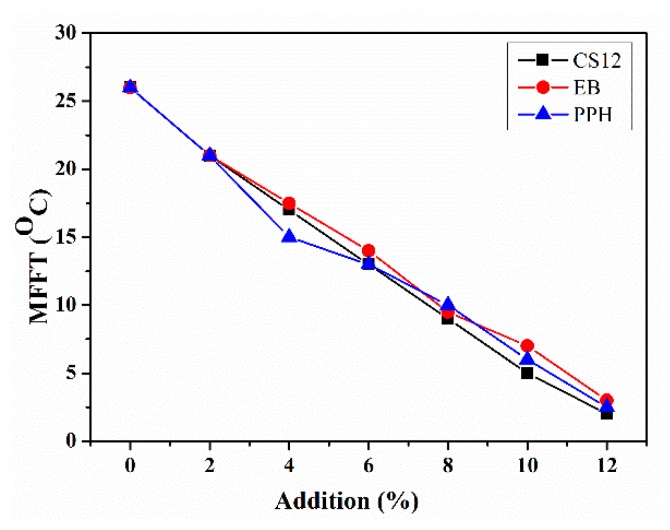
The effect of film-forming additives on the waterborne fluoropolymer. CS12: 2,2,4-trimethylpentanediol monobutyrate; EB: ethylene glycol monobutyl ether; MFFT: minimum film-forming temperature; PPH: propylene glycol phenyl ether.

The film-forming agent is crucial in the process of forming latex films, which further affects the mechanical properties and stability of the coating. Thus, three kinds of film-forming additives were used in the synthesis of fluoropolymer **P1**, and the minimum film-forming temperature was determined as shown in Figure 7. Figure 7 demonstrates that the fluoropolymer emulsion film-forming temperature decreased with an increase in the amount of film-forming agent added, and the degree of reduction was very large. When 8% film-forming agent was added, the film-forming temperature of the resin decreased from 26 °C to 10 °C. The influence of the different types of film-forming additives on the film-forming temperature was virtually identical.

### 3.7. Weatherability of the Waterborne Fluoride Coating

**Figure 8:**
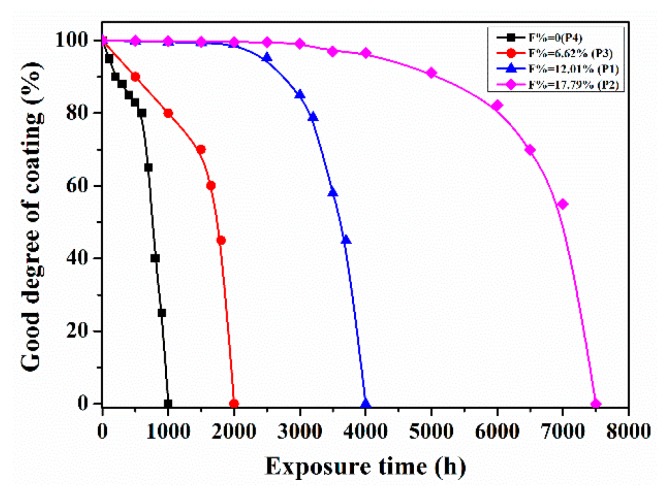
Accelerated weathering test of coatings with different fluoride contents.

The fluoride content of the fluoropolymer has an important influence on the performance of the fluorine coating. The synthesized samples (**P1**, **P2**, **P3**, and **P4**) were used for the preparation of white coatings (fluorine emulsion dosage, 40%), which then underwent the QUV accelerated weathering test. The results are shown in Figure 8. A good degree of coatings in Figure 8 expressed surface change in the coating’s film after aging tests, including pulverization, surface blister, peeling, and crack. The original surface state of the coating’s film was defined as 100%, and when undergoing different exposure times, the coating’s film would show the related destroyed phenomenon. Once severe pulverization, bubbling, or peel-off on the surface of the coating’s film occurred, the surface state was viewed as 0%. The fluoride-free coating (**P4**) only lasted 1000 h in the aging test. Sample **P3** (F = 6.62%) could not pass 2000 h in the aging test. Samples **P1** (F = 12.01%) and **P2** (F = 17.79%) showed pulverization after 4000 h and 7500 h of exposure, respectively. Normally, the shielding effect and steric hindrance effect of fluorine atoms in fluorine-containing polymers provides the copolymer with higher chemical inertness than ordinary polymers. Thus, the fluoropolymer is highly resistant to weather. Low or no fluorine content in the polymer would reduce the protection and shielding effects, and thus weaken weatherability. From the experimental results, the coatings prepared using fluoropolymer with a fluorine content of more than 12 wt % showed excellent weather resistance.

**Figure 9:**
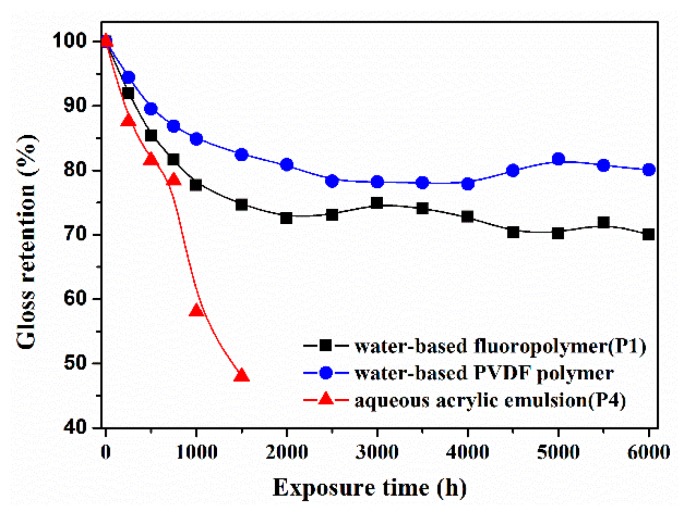
Quick ultraviolet (QUV) tests of different types of waterborne polymer. PVDF: polyvinyldene fluoride.

**Figure 10:**
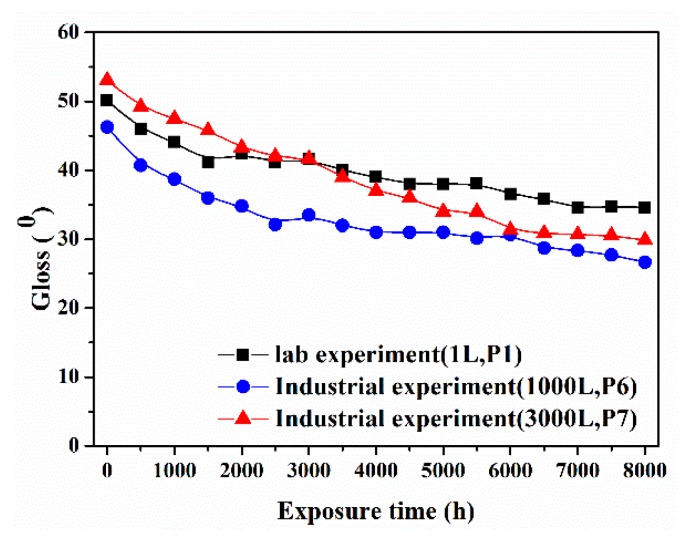
Change in the waterborne fluoropolymer’s gloss over time.

The weatherability of the waterborne fluoropolymer (**P1**) was also compared with that of the waterborne PVDF coating and acrylic coating. To better examine pulverization in the coating, the three resins were blended with titanium dioxide at 5% of the resin weight, which was used to prepare a coating. The QUV accelerated weathering test results of the coating are shown in Figure 9. The weatherability of the waterborne PVDF coating was good, and that of the acrylic emulsion coating was typically poor. The waterborne fluoropolymer (**P1**) coating also showed outstanding weatherability. The scale-up and industrial-scale products of **P6** and **P7** were used for weather resistance testing, and gave the same results as **P1**, as shown in Figure 10. This further demonstrated the reliability and feasibility of using the laboratory-scale formulations and preparation methods.

**Figure 11:**
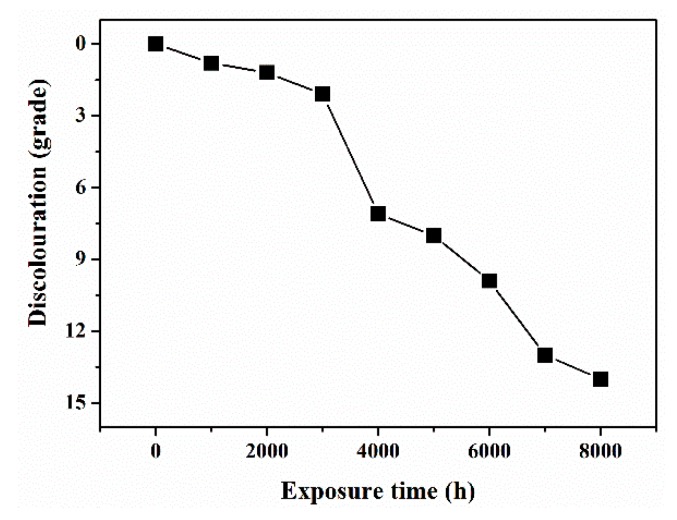
Coating discoloration after the QUV test.

**Figure 12:**
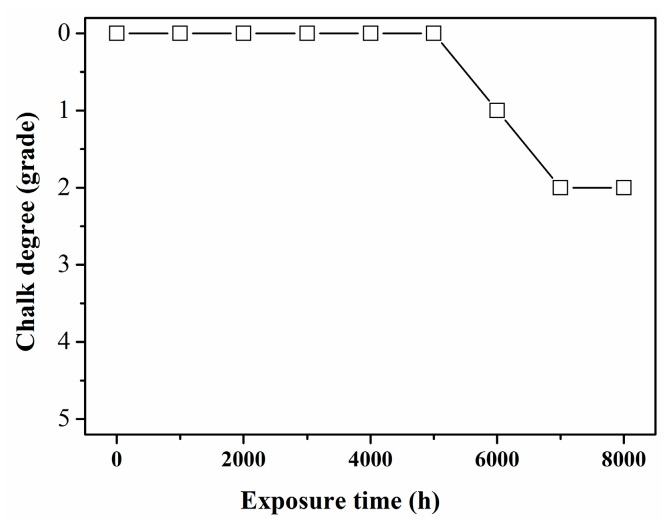
Coating chalk after the QUV test.

A waterborne fluoropolymer blended with a bright-red pigment paste was used in the QUV experiment, where the discoloration and chalk degree of the coating was investigated, as shown in Figure 11 and Figure 12. After 5000 h of exposure, the coating of the sample showed no chalking, but experienced severe discoloration, while the phenomena of bubbling, peel-off, and cracking did not appear.

**Figure 13:**
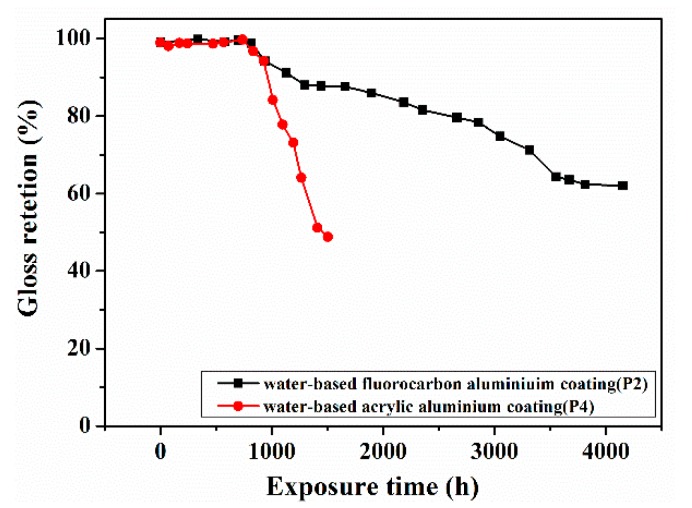
QUV test of coatings of **P2** and **P4** with 5% aluminum powder.

Aqueous aluminum powder coatings were prepared using the waterborne fluoropolymer **P2** and the acrylic emulsion **P4**, and underwent weatherability testing, as shown in Figure 13. The results indicated that the waterborne fluoropolymer was more suitable for the preparation of a waterborne aluminum coating than the acrylic emulsion.

## 4. Conclusions

Waterborne fluoropolymers were synthesized using CTFE, VAc, BA, Veova 10, and AA using the semi-continuous emulsion polymerization approach. Its reliability and feasibility were verified by scale-up and industrial-scale experiments.

The waterborne fluoropolymers had conversions of more than 96% and coagulum amounts of 0.01%–0.1%. The appearance had a slight blue tint, and they showed good stability, narrow particle diameter distributions, and average particle diameters of 100–200 nm. The results of FT-IR, the fluoride content, and the QUV accelerated weathering test showed that CTFE was effectively and uniformly involved in the copolymerization, and the thermal stability of waterborne fluoropolymer was improved.

The accelerated weathering test indicated that the weatherability of waterborne fluoropolymers with more than 12% fluoride content was satisfactory. From an economical viewpoint, waterborne fluoropolymers with 12% fluoride content proved to be a promising category of materials and a good choice for super-weatherable coatings, as fluoride monomers are expensive.

## Figures and Tables

**Table 1:** Amounts used in the emulsion polymerization of **P1**.

	CTFE	VAc	Veova-10	BA	AA	NP-10	600#A	SDS	KPS	NaHCO_3_	H_2_O	AMP-95
Charge (g)	85.00	8.74	2.91	2.91	0.23	1.56	1.44	0.34	0.35	0.90	212.19	
Feed (g)	0	141.24	47.09	47.09	3.67	4.36	4.05	0.93	1.16	0	163.72	
Addition (g)	0	0	0	0	0	0	0	0	0.35	0	18.25	4.88
Total (g)	85.00	149.98	50.00	50.00	3.90	5.92	5.49	1.27	1.86	0.90	394.16	4.88

**Table 2:** Results of the emulsion polymerizations.

Polymer	Conversion (%)	Coagulum (wt %)	Non-Volatile (wt %)	Appearance	F (wt %)	Average Particle Diameter (nm)	Molecular Weight (Mn)	Dispersities (Mw/Mn)
**P1**	99.34	0.01	44.89	milky white	12.01	163	29,221	8.30
**P2**	96.73	0.07	44.26	milky white	17.79	171	40,300	14.3
**P3**	98.93	0.05	43.54	milky white	6.62	165	no data	—
**P4**	97.39	0.10	36.19	milky white	0	193	no data	—
**P5**	97.57	0.07	44.40	milky white	12.05	101	42,636	5.95
**P6**	98.20	0.05	46.82	milky white	12.08	176	28,900	13.8
**P7**	99.18	0.04	42.60	milky white	12.12	181	51,233	9.74
